# Microbial Clearance of Refractory *Mycobacterium avium* Complex Pulmonary Disease During Pembrolizumab and Intensified Antimicrobial Therapy: A Case Report

**DOI:** 10.3390/ph18101464

**Published:** 2025-09-29

**Authors:** Jan Naseer Kaur, Parikshit Padhi, Suhani Pal

**Affiliations:** 1 Department of Pharmacy Practice, School of Pharmacy and Pharmaceutical Sciences, University at Buffalo, Buffalo, NY 14215, USA; 2 Department of Internal Medicine, Jacobs School of Medicine & Biomedical Sciences, University at Buffalo, Buffalo, NY 14203, USA; 3 Nichols School, Buffalo, Buffalo, NY 14216, USA

**Keywords:** *Mycobacterium avium* complex, drug resistance, pembrolizumab, immune checkpoint inhibitors, host-directed therapy

## Abstract

**Background:** Pulmonary infections caused by *Mycobacterium avium* complex (MAC) are notoriously difficult to treat, particularly in the context of multidrug resistance. Immunotherapy with checkpoint inhibitors, though effective in various malignancies, has uncertain immunomodulatory effects on chronic infections such as MAC. **Case Summary:** We report a case of a 67-year-old man with at least a 6-year history of treatment refractory MAC who developed stage IIIB mismatch repair-deficient colon adenocarcinoma. Despite years of multidrug therapy, the patient had persistent positive acid-fast bacilli cultures. Upon initiation of pembrolizumab for colon cancer, with no concurrent anti-MAC therapy, he experienced radiologic and microbiologic improvement. Following subsequent reinitiation of multidrug MAC therapy, he achieved his first documented negative culture after over a decade of infection and has remained culture-negative and in cancer remission for over two years. **Conclusions:** This case suggests that immune checkpoint inhibitors like pembrolizumab may enhance antimicrobial immune responses against MAC through PD-1 pathway modulation. The temporal association between pembrolizumab therapy and MAC clearance warrants further investigation into immunomodulatory approaches for drug-resistant mycobacterial infections, particularly as adjunctive therapy to conventional antimicrobial regimens.

## 1. Introduction

Nontuberculous mycobacteria (NTM), particularly *Mycobacterium avium* complex (MAC), have emerged as significant pulmonary pathogens in immunocompetent and immunocompromised populations alike [1]. The management of MAC pulmonary disease is particularly challenging due to its protracted clinical course, variable drug susceptibility, and high rates of relapse or treatment failure [2]. Treatment typically includes macrolides, ethambutol, and rifampicins, yet given the emergence of drug resistance, especially to clarithromycin, the backbone of therapy is associated with poor outcomes [1,3,4]. The intersection of MAC pulmonary disease with malignancy, particularly when requiring immune checkpoint inhibitors like pembrolizumab, presents a unique therapeutic challenge. There is limited research addressing how immunotherapy may influence chronic infections, particularly when co-administered with multidrug antimicrobial regimens. We present a rare case of persistent, treatment-refractory MAC pulmonary disease complicated by colon adenocarcinoma managed with pembrolizumab, after which the patient exhibited unexpected microbiologic clearance of MAC.

## 2. Case Presentation

We report the case of a 67-year-old male with chronic obstructive pulmonary disease and significant tobacco use who was first diagnosed with pulmonary *Mycobacterium avium* complex (MAC) in 2008, following the identification of MAC in a sputum culture. Unfortunately, he did not follow up on treatment. In late 2014, he re-presented with worsening respiratory symptoms such as cough and dyspnea on exertion. Computed tomography (CT) of the chest revealed multiple cavitary lesions in the right upper and lower lobes of the lung, with the largest measuring 5.5 cm in the right upper lobe. Bronchoscopy ruled out malignancy, but acid-fast bacilli (AFB) cultures confirmed MAC with susceptibility to rifampin, ethambutol, clarithromycin, and amikacin; linezolid and moxifloxacin exhibited intermediate activity.

**Figure 1:**
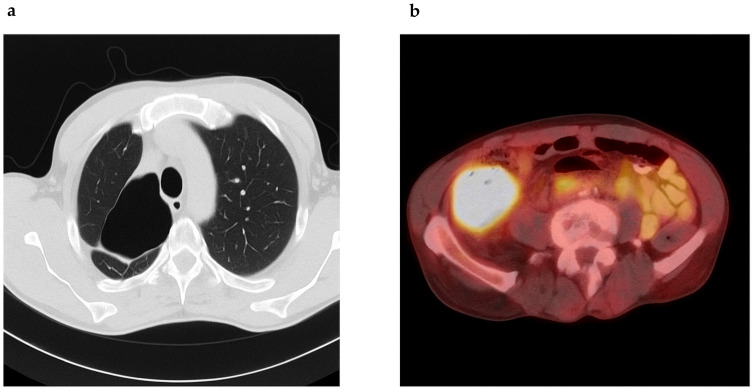
(**a**) Chest CT reveals a thick-walled cavitary lesion in the apex of the right lung measuring approximately 8.2 × 7.4 cm with other blebs noted in the right upper lobe. (**b**) PET scan reveals a 7.4 cm transaxial markedly hypermetabolic mass in the ascending colon with SUV max 26.0.

**Figure 2:**
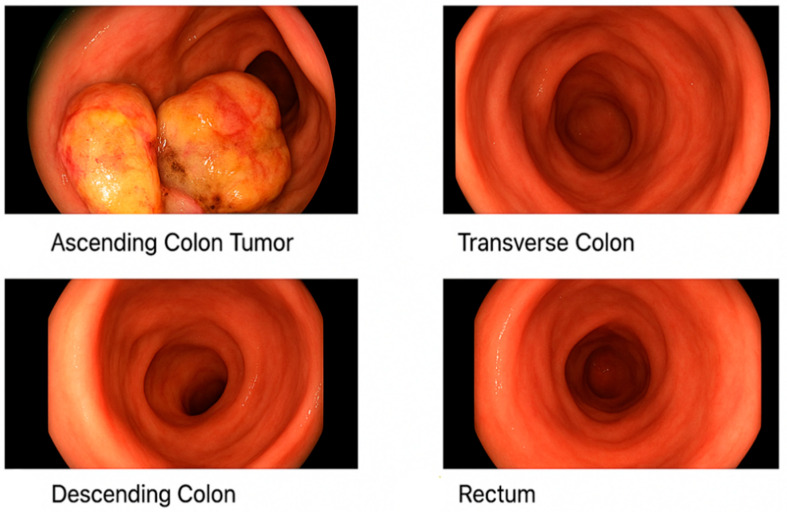
A fungating, infiltrative, polypoid sessile, submucosal, and ulcerated partially obstructing large mass is seen in the ascending colon (**top left**). The transverse colon (**top right**), descending colon (**bottom left**) and rectum (**bottom right**) are all normal. Biopsy from the ascending colon mass revealed colon adenocarcinoma.

He was initiated on a regimen of clarithromycin, ethambutol, and rifampin. Despite some clinical improvement, sputum cultures remained persistently positive. In 2016, moxifloxacin was added to intensify therapy. By 2019, he experienced worsening symptoms, and drug susceptibility testing revealed resistance to clarithromycin, amikacin, ciprofloxacin, and linezolid, suggestive of treatment-emergent resistance, while retaining sensitivity to ethambutol, rifampin, and clofazimine (Table 1). He was treated with ethambutol, rifampin, azithromycin, and clofazimine (compassionate use), though clofazimine had to be discontinued due to insurance constraints. He declined inhaled aminoglycosides. Eventually, due to gastrointestinal intolerance and persistent symptoms, he self-discontinued therapy in early 2022. Chest CT in mid-2022 revealed progression, with cavitary lesions measuring up to 8.2 × 7.4 cm (Figure 1a). Sputum AFB remained positive, with drug susceptibility revealing resistance to clarithromycin and moxifloxacin, but susceptibility to bedaquiline, omadacycline, tedizolid, and inhaled liposomal amikacin. Simultaneously, workup for his chronic abdominal pain revealed a cecal mass. Colonoscopy confirmed a fungating mass in the cecum/ascending colon and biopsy of the mass confirmed a moderately differentiated adenocarcinoma with loss of MutL promoter homolog 1 (MLH1) and postmeiotic segregation gene 2 (PMS2), suggestive of mismatch repair deficiency (MMR-d) (Figure 2). Lynch syndrome was ruled out by genetic testing. A positron emission tomography (PET)-CT scan showed a hypermetabolic ascending colon mass and multiple fludeoxyglucose (FDG)-avid abdominal lymph nodes (Figure 1b). Lung cavities were also PET-avid. Hence, we performed a lung biopsy, which came back negative for malignancy. His baseline carcinoembryonic antigen (CEA) was at 138.2 ng/mL (normal < 4 ng/mL).

**Figure 3:**
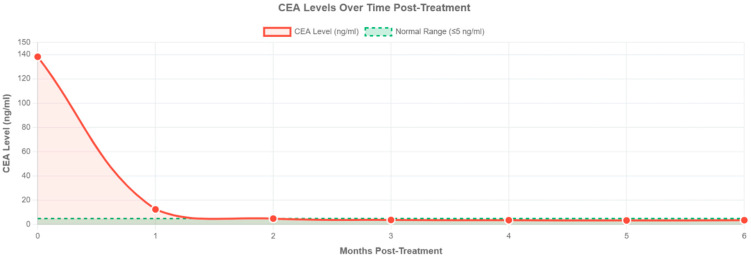
CEA levels following treatment over 6 months. CEA concentrations (ng/mL) were measured at monthly intervals post-treatment. The dashed green line indicates the upper limit of normal (4 ng/mL). CEA decreased from 138.2 ng/mL at baseline to 3.6 ng/ml in 6 months (97.4% reduction), with normalization achieved by month 2.

Given his inoperable stage IIIB colon cancer and MMR-d status, he was initiated on neoadjuvant pembrolizumab (200 mg every 3 weeks). Notably, he was off MAC therapy at this time. After four cycles of pembrolizumab, his CEA levels had normalized (Figure 3). A repeat CT scan after 4 cycles showed marked reduction in tumor burden and nodal involvement. Due to symptomatic improvement and persistently positive AFB cultures, MAC therapy was reinitiated with bedaquiline, clofazimine, ethambutol, rifampin, and both IV and inhaled liposomal amikacin. He completed eight cycles of pembrolizumab by May 2023. Follow-up imaging showed complete radiologic response of the colon mass, and right hemicolectomy revealed only residual microscopic disease (ypT1, ypN0, 0/17 lymph nodes). He remains in remission under surveillance. In May 2023, he was found to have a negative AFB culture in his sputum. This was the first time since his diagnosis of MAC that he had negative cultures. This was repeated again in 6 months, and the mycobacterial cultures were negative. Despite his inconsistency in adherence to anti-MAC treatment as of mid-2025, his AFB cultures have consistently turned negative. Ever since he received pembrolizumab, he has remained in remission from both MAC and colorectal cancer (Supplementary Figure S1).

## 3. Discussion

This case highlights the diagnostic and therapeutic complexities of refractory MAC pulmonary disease, particularly in the setting of evolving drug resistance. Over more than a decade, this patient developed resistance to multiple cornerstone agents, including clarithromycin and amikacin, rendering standard therapy ineffective. Notably, treatment options like bedaquiline and clofazimine, traditionally reserved for multidrug-resistant tuberculosis, became necessary and reflect an emerging strategy for drug-resistant NTM.

The patient’s course was further complicated by the unexpected diagnosis of MSI-H colon adenocarcinoma, which necessitated immune checkpoint inhibitor (ICI) with pembrolizumab. While ICIs are not traditionally contraindicated in chronic infections, their role in modulating host–pathogen dynamics is still under investigation. Some reports suggest that PD-1 blockade may enhance antimicrobial immune responses, though data in NTM infections are sparse. ICIs are now used in many cancers such as melanoma, non-small-cell lung cancer, small-cell lung cancer, kidney cancers, gastrointestinal malignancies, and triple-negative breast cancers, to name a few. These include cytotoxic T-lymphocyte antigen 4 (CTLA-4) inhibitors such as ipilimumab, programmed cell death protein 1 (PD1) inhibitors such as pembrolizumab and nivolumab, and programmed cell death ligand 1 (PDL1) inhibitors such as atezolizumab and durvalumab. PD-1 engagement reduces the cytokine release of, for example, interleukin-2, interferon-ϒ (IFN-ϒ), and TNF-α, as well as cell proliferation through interference with the CD28-costimulatory pathway [6]. Pembrolizumab is a humanized IgG4 monoclonal antibody targeting the PD-1/PDL-1 pathway leading to the inhibition of immune suppression and T-cell activation [7]. Based on the Keynote-177 trial, pembrolizumab was approved in MSI-H advanced colorectal cancers [8].

We know that in the past, disseminated MAC infection used to occur in immunocompromised patients, such as those with HIV, especially if their CD4 count is less than 100 [9]. Grunberg et al. mentioned the role of interleukin-12 (IL-12) as an important cytokine in the immune response against MAC, and in patients with HIV, there is downregulation of IL-12 and TNF-α [10]. In mycobacterium tuberculosis, T-cells such as Th1 and Th2 cells in conjunction with cytokines such as TNF-α and IFN-ϒ play a role in controlling this infection [11]. When an organism becomes persistent in the body, such as with MAC infection, it can evade the hosts’ immune system. Mycobacterial infections such as TB can modulate the immune response by downregulating T-cells and can constantly stimulate certain CD4+ T-cells, leading to immune exhaustion [12]. MAC is also able to escape the immune system. One theory is that MAC can negate LAMP1 when phagocytosed by macrophages, which in turn leads to a decrease in cytokine release and leads to compartmentalization of the bacteria [13]. Shu et al. evaluated the interleukin levels in patients with MAC lung disease. Their research showed that the levels of IL-17 were lower in MAC lung disease patients due to an increase in PD-1 levels, and by blocking PD-1 and PDL-1, they were able to increase IL-17 levels [14]. This shows that PD-1 and PDL-1 play a role in immune evasion in MAC infections, and hence, anti-PD-1 and anti-PDL-1 medications may inadvertently stimulate the immune response against these chronic infections as well. This still needs to be studied, as mouse models have shown that antiPD-1 medications may make them more susceptible to tuberculosis [15]. Mejia-Chew et al. studied IL-7 injections in patients with MDR TB, and of the six patients who completed the course of IL-7 injections, none were able to be converted to sputum-negative [16].

A striking observation in this case is the sustained negativity of persistently positive AFB cultures to negative, following the combined use of pembrolizumab and intensified multidrug MAC therapy. Whether pembrolizumab had a direct or immunomodulatory effect on MAC control is speculative, but plausible given emerging evidence of PD-1 pathway involvement in immune evasion by mycobacteria. Furthermore, the timing of sputum being negative following long-term treatment failure raises the possibility that immune checkpoint blockade played an adjunctive role in overcoming bacterial persistence. This case underscores the need for further investigation into the interplay between chronic mycobacterial infections and cancer immunotherapy, particularly in patients with dual diagnoses requiring overlapping treatments.

The emergence of multidrug-resistant MAC infections has necessitated the exploration of novel therapeutic agents traditionally reserved for tuberculosis treatment, as well as innovative immunomodulatory approaches. Bedaquiline, a diarylquinolone antibiotic, has demonstrated low resistance rates against MAC isolates and shows promise as a salvage therapy for treatment-refractory cases, while newer agents such as tedizolid and omadacycline have shown low resistance rates against MAC isolates [17]. In vitro studies suggest that bedaquiline/clofazimine combination regimens may add significant activity to MAC treatment, with the bedaquiline/clofazimine combination showing superior efficacy compared to bedaquiline alone [18]. Beyond antimicrobial therapy, host-directed therapy (HDT) has emerged as a promising approach that aims to interfere with host cell factors required by pathogens for replication or persistence, while enhancing protective immune responses against the pathogen [19,20]. MAC represents an increasingly important cause of morbidity and mortality, and host-directed therapies are being investigated as potential adjunctive treatments [5]. The case presented here suggests that immune checkpoint inhibitors like pembrolizumab may inadvertently enhance antimicrobial immune responses against MAC, as evidenced by the patient’s culture reports from persistently positive to negative sputum cultures following pembrolizumab therapy. Research reveals conflicting data regarding the impact of ICIs on mycobacterial disease outcomes. While some studies demonstrate that corticosteroid administration for managing ICI-related immune adverse effects has led to tuberculosis reactivation, emerging evidence suggests that ICIs may also facilitate complete remission of MAC infections [21,22]. Given the continuing challenges posed by infectious diseases and the need for newer approaches to improve treatment outcomes, the combination of novel antimicrobial agents with immunomodulatory strategies represents a promising frontier in managing drug-resistant MAC infections

## Figures and Tables

**Table 1:** Antimicrobial susceptibility results for *Mycobacterium avium* complex (2015–2023).

Antimicrobial Agent/Test	Feb 2015	Aug 2016	Jun 2021	Jan 2022	Oct 2022	May 2023
**Amikacin**	4 (S)	16 (S)	32 (I)	32 (I)	16 (S)	—
**Clarithromycin**	2 (S)	2 (S)	>64 (R)	>64 (R)	>64 (R)	—
**Linezolid**	16 (I)	16 (I)	16 (I)	16 (I)	16 (I)	—
**Moxifloxacin**	2 (I)	4 (R)	4 (R)	4 (R)	>4 (R)	—
**Rifabutin**	≤0.25 (NI)	≤0.25 (NI)	—	—	2	—
**Ethambutol**	≤0.5 (NI)	16 (NI)	—	—	—	—
**Streptomycin**	—	—	64 (NI)	—	>32	—
**Omadacycline**	—	—	—	—	>8	—
**Bedaquiline**	—	—	—	—	0.004	—
**Tedizolid**	—	—	—	—	4	—
**Ciprofloxacin**	—	—	—	—	>8	—
**Doxycycline**	—	—	—	—	>8	—
**Minocycline**	—	—	—	—	>8	—
**Rifampin**	—	—	—	—	>4	—
**Trimethoprim** **/Sulfamethoxazole**	—	—	—	—	2/38	—
**AFB Culture**	MAC isolated	MAC isolated	MAC isolated	MAC isolated	MAC isolated	Negative

All MICs (Minimum Inhibitory Concentrations) are expressed in µg/mL. Susceptibility testing was performed via broth microdilution (where CLSI breakpoints exist for MAC). S = susceptible; R = resistant; I = intermediate; NI = no interpretive category for MAC; — = Test not performed; MIC provided for reference only. Bedaquiline MIC reference: Brown-Elliott BA et al., AAC 2017 [5]; typical reported range of 0.002–0.06 mg/L for MAC isolates.

## Data Availability

The original contributions presented in this study are included in the article and Supplementary Material. Further inquiries can be directed to the corresponding authors.

## Supplementary Materials

The following is available online at https://www.mdpi.com/article/10.3390/ph18101464/s1, Figure S1: Clinical timeline of a patient with *Mycobacterium avium* complex (MAC) lung disease and colorectal cancer.
