# Supplementary material for: Microbial Clearance of Refractory Mycobacterium avium Complex Pulmonary Disease During Pembrolizumab and Intensified Antimicrobial Therapy: A Case Report
Source: Pharmaceuticals (Basel). 2025 Sep 29;18(10):1464. doi: 10.3390/ph18101464 (PMC12567303; doi:10.3390/ph18101464)
Supplement: Supplementary file 1 [file pharmaceuticals-18-01464-s001.zip › pharmaceuticals-3842144-supplementary.pdf]

Supplementary Materials

Supplementary Figure S1.

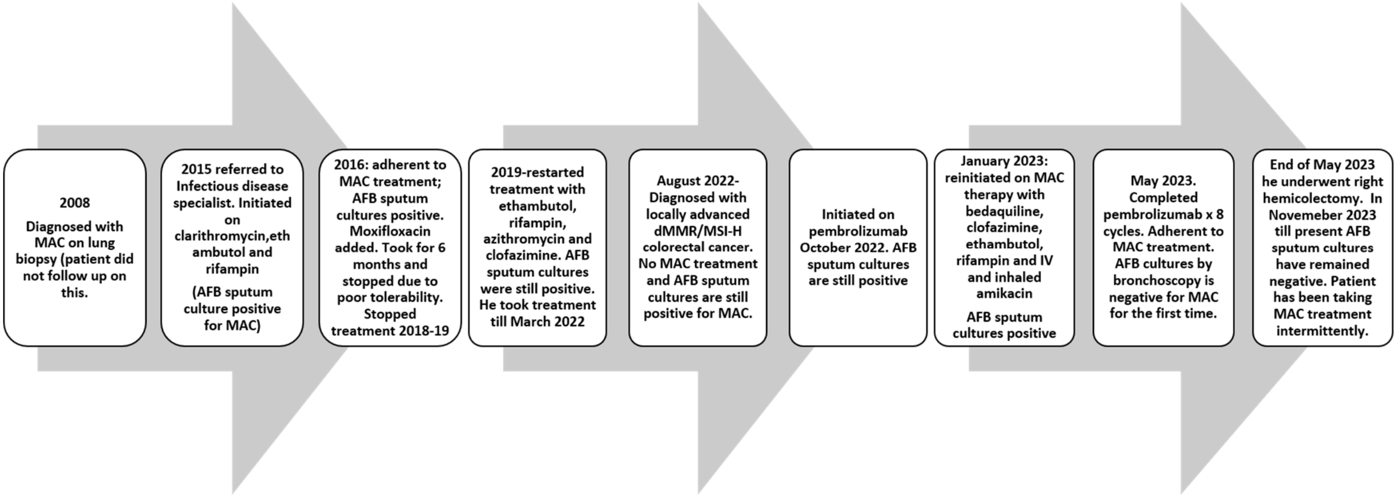

Scheme 1. Clinical timeline of a patient with Mycobacterium avium complex (MAC) lung disease and colorectal cancer.
The timeline outlines diagnosis and sequential treatments for MAC lung disease from 2008 to 2023, including multiple antibiotic regimens and pembrolizumab therapy.
